# Results of treatment of acute occlusions of limb arteries at a university hospital - retrospective study

**DOI:** 10.1590/1677-5449.200031

**Published:** 2020-11-16

**Authors:** Caroline Teodoro, Matheus Bertanha, Flavia Potsch Camara Mattos Girard, Marcone Lima Sobreira, Ricardo de Alvarenga Yoshida, Regina Moura, Rodrigo Gibin Jaldin, Winston Bonetti Yoshida

**Affiliations:** 1 Cirurgia e Ortopedia, Universidade Estadual Paulista “Júlio de Mesquita Filho” (UNESP), Botucatu, SP, Brasil.

**Keywords:** Balloon Embolectomy, Ischemia, Lower Extremity, Upper Extremity

## Abstract

**Background:**

Acute arterial occlusions (AAO) in limbs have been increasing in parallel with population longevity.

**Objective:**

To assess risk factors, limb salvage rates, and survival of patients with AAO treated at a University Hospital.

**Methods:**

Retrospective cohort study of consecutive patients. Outcomes included: patency, symptoms, comorbidities, Rutherford category, arteries occluded, postoperative complications, and 30-day limb salvage and mortality rates.

**Results:**

Medical records were evaluated from 105 patients, predominantly males (65.7%), with ages ranging from 46 to 91 years. Etiology: thrombotic (54.3%), embolic (35.2%), and undefined (10.5%). About 2/3 of the patients were assessed as Rutherford category II or III. Associated symptoms: pain (97.1%), coldness (89.5%), pallor (64.7%), sensory loss (44.7%), paralysis (30.5%), anesthesia (21.9%), edema (21.9%), and cyanosis (15.2%). Associated comorbidities: hypertension (65.0%), smoking (59.0%), arrhythmias (26.6%), dyslipidemia (24.0%), and diabetes (23.8%). The distal superficial femoral-popliteal segment was the most affected (80%). Thromboembolectomy with a Fogarty catheter was performed in 73.3% of cases (81.0% of embolic cases, 71.9% of thrombotic cases, and 54.5% of cases with undefined etiology) and was the only treatment used in 41 cases (39.05%), among which there were 11 reocclusion, 20 amputations, and 14 deaths. Arterial reocclusion was more frequent in thrombosis cases (12.9%, p = 0.054). Within 30 days of treatment, total mortality was 14.6%, and 19.8% of cases underwent major amputation, which was less frequent among Rutherford Class I patients (p = 0.0179).

**Conclusion:**

Treatment of AAO was primarily performed by thromboembolectomy with a Fogarty catheter, either alone or in combination with other treatments, achieving amputation and complication rates compatible with the best results in the literature and were progressively lower in less advanced Rutherford categories.

## INTRODUCTION

An acute arterial occlusion (AAO) is defined as a sudden fall in blood perfusion that threatens the viability of a limb.^1^ In general, the painful symptoms are abrupt and vary from sudden onset intermittent claudication or worsened claudication, to pain at rest, coldness (poikilothermia), sensory loss, and muscle weakness or paralysis. On physical examination, pulses will be absent distal of the site of occlusion, with coldness, pallor, or cyanosis of the skin and loss of sensitivity in the affected limb.^2^ Without swift and timely vascular intervention, in the majority of cases, progression is associated with poor prognosis for limb salvage, since irreversible limb ischemia can set in, requiring amputations.

Incidence varies in the range of 14/100,000 to 17/100,000 inhabitants or 1.5 cases per 10,000 people per year^1^^,^^3^^,^^4^ and has been growing as longevity of the population increases. The most frequent causes are emboli caused by cardiac or aortic disorders and thromboses from atherosclerotic plaques, which are consequences of atherothrombotic complications in peripheral arteries or occlusions of arterial restorations, or of traumas in patients without atherosclerosis, especially iatrogenic trauma.^3^

Blood flow can be restored using endovascular techniques (mechanical or pharmacological thrombolysis, angioplasty, and stenting) or by open surgery, such as thromboembolectomy with Fogarty catheter, bypass, endarterectomy with or without arterial patching, or hybrid techniques.^1^ Patients with femoral and popliteal emboli have higher amputation rates, directly related to the time elapsed from occlusion to treatment.^5^ In cases of arterial thromboses related to peripheral arterial disease (PAD), treatment of underlying complicated atheromatous plaques responsible for the thrombus is fundamental to sustained success of revascularization.

One of the most widely used and proven treatment methods is thromboembolectomy with a balloon catheter, which was introduced in 1963 by Fogarty et al.^6^ and is more effective with emboli than for cases with other etiologies.^1^^,^^7^ While these techniques have revolutionized treatment of AAOs, many complications still occur and amputation rates vary from approximately 6 to 30%, with mortality in the range of 18 to 25%.^1^^,^^3^

There is little information on results and outcomes of patients with AAO treated by the Brazilian National Health Service (SUS - Sistema Único de Saúde) in Brazil. Studies of use of fibrinolytics^7^^-^10 predominate in the Brazilian literature, but this treatment is generally not available in hospitals affiliated to the SUS. There are no Brazilian studies whatsoever in the literature reporting the results of systematic and use of thromboembolectomy with Fogarty catheters as first-choice treatment for AAOs of the extremities, irrespective of etiology, which is the reason this study was conducted.

## OBJECTIVE

The objective of this study was to retrospectively assess cases of AAO treated at a high complexity SUS cardiovascular service at a public university hospital, analyzing risk factors, limb salvage results, and perioperative survival of patients.

## METHODS

This is a retrospective cohort study of a consecutive series of cases. Patients were included who had been diagnosed with AAO in lower or upper limbs and treated between 2012 and 2017, selected by analysis of data from electronic patient records at a single university hospital. Patients were excluded if they were under the age of 18 or were pregnant, had occlusions of arterial bypasses, cancer, or arterites, or if their medical records were incomplete. This project was approved by the local Research Ethics Committee (Decision number 737,804).

In all cases, a dedicated AAO chart was filled out prospectively and any missing data were completed by retrospective review of medical records. Demographic data, risk factors, signs and symptoms, and Rutherford clinical categories for classification of the degree of ischemia in the limb involved (Society of Vascular Surgery/The International Society of Cardiovascular Surgery classification)^11^ were tabulated (Table 1), in addition to treatments administered and the main complications (amputations, reocclusions, need for fasciotomy, renal failure, pulmonary and cardiac complications, amputations, and operative mortality).

**Table 1:** Clinical classification according to progression of ischemia in the limb involved, adapted from Rutherford (Society of Vascular Surgery/The International Society of Cardiovascular Surgery classification).^3^

**Classification**	**Clinical signs and Doppler findings**
Classification I – Viable limb	Absence of neurological signs, arterial sound audible on Doppler.
Classification II – Viability threatened – reversible ischemia	IIa – Marginally threatened: sensory loss, arterial sound inaudible on Doppler, venous sound audible.
	IIb – Immediately threatened: some degree of muscle weakness, arterial sound inaudible on Doppler, venous sound audible.
Classification III – Irreversible ischemia	Paralysis, contracture, arterial and venous sound inaudible on Doppler.

Patients were divided into three groups, according to AAO etiology: group 1 – emboli; group 2 – thromboses; and group 3 – undefined. The following factors were considered when allocating patients: acute or gradual onset; intense or moderate pain; prior intermittent claudication present or absent; cardiac arrhythmia present or absent; contralateral pulses present or absent; and intraoperative findings compatible with emboli or thrombosis.^1^ Patients were classified as undefined if they did not fit the characteristic criteria for emboli or for thrombosis.

The treatment protocol followed at the hospital is to prioritize thromboembolectomy with a Fogarty catheter for immediate relief of acute ischemia, for both emboli and thrombosis cases. The preferred approach for surgical access is dissection and exposure of the common femoral, superficial femoral, or brachial arteries, depending on the case and supplemented by distal accesses when needed. The protocol includes routine use of intraoperative arteriography after thromboembolectomy to evaluate results and to identify other strategies during surgery.

In embolic cases, unfractionated heparin (UFH) was used during the postoperative period to prevent recurrence. In thrombotic cases treated using endovascular procedures or bypasses, double platelet antiaggregation was prescribed. Thromboembolectomy with a Fogarty catheter was indicated in some Rutherford III cases to relieve ischemia and/or restore blood flow to collateral arteries, with the objective of achieving a more distal amputation and/or osteoarticular preservation, for better rehabilitation. Primary outcomes were: death, arterial reocclusion, and need for major amputation (transtibial and/or transfemoral or of an upper limb).

## PATIENTS

Sample selection was by convenience, analyzing all consecutive cases that met the inclusion criteria during the study period. On the basis of data in the literature indicating from 18 to 25% mortality (considering a median value of 22%), and expecting this rate to fall to 15%, the sample size was calculated at 135 patients, with 80% test power and a 5% significance level.

## STATISTICAL ANALYSIS

Descriptive statistics were calculated for quantitative variables, which were then stratified by final diagnosis (emboli, thrombosis, or undefined). Comparisons between means per diagnosis were made using analysis of variance (ANOVA), followed by the Tukey test. Comparisons between preoperative and postoperative continuous data were made using Student’s *t* test for paired samples, per diagnosis. For categorical variables, associations with final diagnosis were analyzed using the chi-square test or Fisher’s test. The significance level was set at 5% or the equivalent *p*-value. All analyses were performed using SAS for Windows, v.9.4 (SAS Institute Inc. North Carolina, United States).

## RESULTS

**Figure 1:**
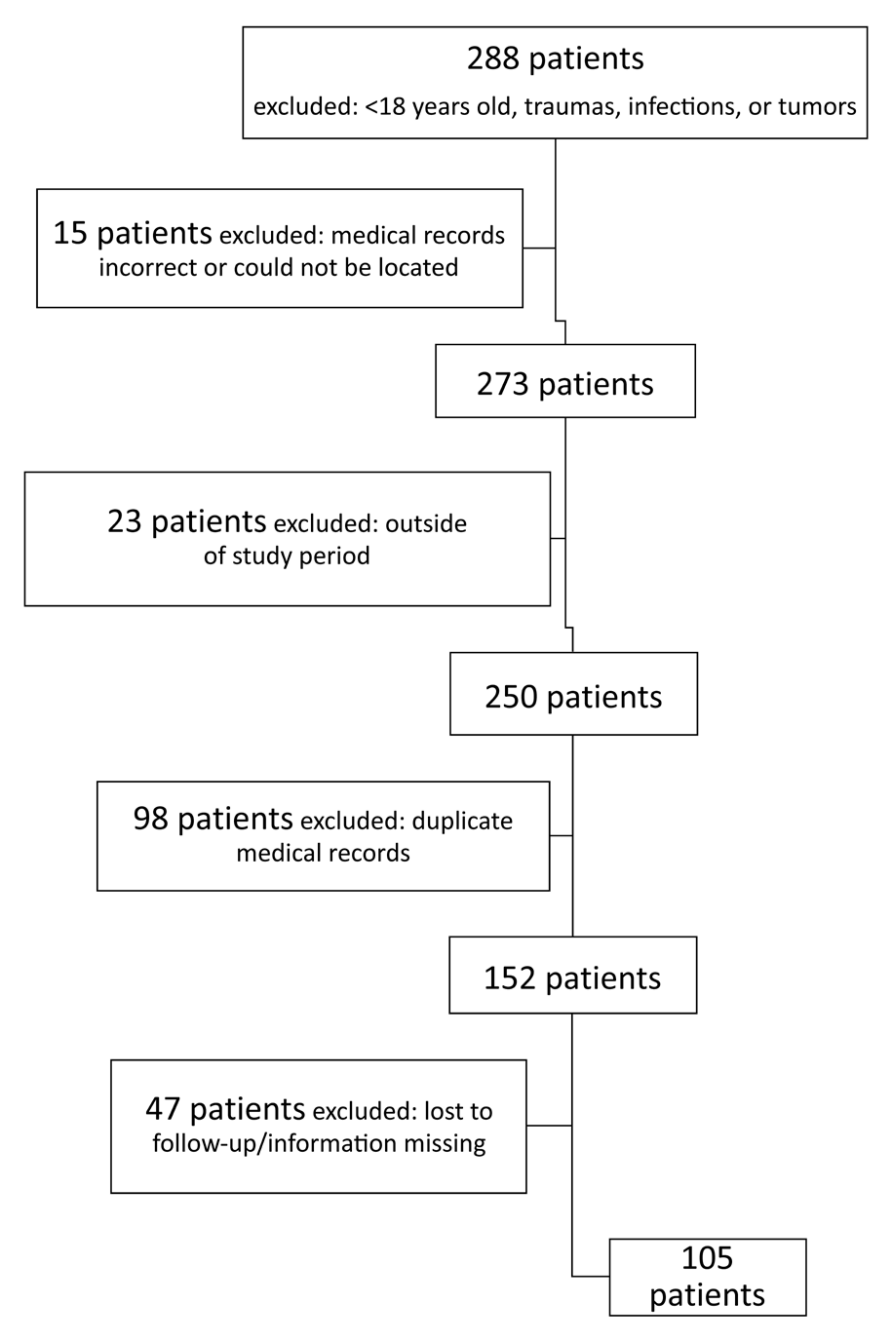
Flow diagram illustrating inclusion and exclusion of patients for the study.

One hundred and eighty-three of the 288 records of AAO found were excluded because of factors that are detailed in Figure 1. Therefore, a total of 105 patient records were analyzed. Table 2 shows demographic data and outcomes according to etiology (per group). Thrombosis was the most prevalent etiology (54.3%), followed by embolic etiology (35.2%) and then undefined etiology (10.5%), with no difference between the sexes. White skin color was the most frequent (85.7%) and occlusions were in lower limbs in the majority of cases (89.5%). Rutherford classification III had the smallest number of patients in absolute terms, but with no statistical difference between groups. There were no statistical differences between groups in terms of the outcomes reocclusion, amputation, or death within 30 days. Atrial fibrillation (AF) was statistically more frequent in group 1 – emboli (p = 0.0001), and popliteal artery aneurysm, PAD, and smoking were all statistically more frequent in group 2 – thrombosis (p = 0.0138, p = 0.0125, and p = 0.0161, respectively).

**Table 2:** Demographic data and main outcomes, by etiology of acute arterial occlusion (AAO).

		**Emboli (n = 37)**	**Thrombosis (n = 57)**	**Undefined (n = 11)**	**Total (n = 105)**	**p**
Age (years)	Maximum	91	87	90	91	-
	Minimum	48	47	52	46	-
	Mean	70	69	73	69.5	-
Sex	Male	25 (23.81%)	37 (35.24%)	7 (6.67%)	69 (65.71%)	0.9542
	Female	12 (11.43%)	20 (19.05%)	4 (3.81%)	36 (34.29%)	
Ethnicity	Caucasian	28 (26.67%)	53 (50.48%)	9 (8.57%)	90 (85.7%)	0.0164
	Other	9 (8.56%)	4 (3.81%)	2 (1.9%)	15 (14.3%)	
Limb	RUL	4 (3.81%)	0	1 (0.95%)	5 (4.76%)	
	LUL	2 (1.9%)	1 (0.95%)	1 (0.95%)	4 (3.81%)	
	RLL	13 (12.38%)	19 (18.1%)	7 (6.67%)	39 (37.14%)	
	LLL	16 (15.24%)	36 (34.29%)	2 (2.86%)	55 (52.38%)	
	Both lower limbs	2 (1.9%)	0	0	2 (1.9%)	
Rutherford classification^11^	I	12 (11.43%)	19 (18.1%)	4 (3.81%)	35 (33.3%)	0.9709
	IIa	11 (10.48%)	15 (14.29%)	2 (1.9%)	28 (26.7%)	0.7460
	IIb	11 (10.48%)	15 (14.29%)	2 (1.9%)	28 (26.7%)	0.7460
	III	3 (2.86%)	8 (7.62%)	3 (2.86%)	14 (13.3%)	0.2531
Reocclusion		4 (3.81%)	13 (12.38%)	0	17 (16.19%)	0.0546
Primary amputation		10 (9.52%)	12 (11.43%)	4 (3.81%)	26 (24.76%)	0.5175
30-day mortality		6 (5.71%)	7 (6.67%)	1 (0.95%)	14 (13.34%)	0.7877
Risk factors	Popliteal aneurysm	0	9 (8.6%)	3 (2.9%)	12 (11.4%)	0.0138
	PAD	7 (6.7%)	28 (26.7%)	4 (3.8%)	39 (37.1%)	0.0125
	Smoking	15 (14.3%)	40 (38.1%)	7 (6.7%)	62 (59.0%)	0.0161
	AF	19 (18.1%)	1 (0.95%)	1 (0.95%)	21 (20%)	0.0001

RUL: right upper limb; LUL: left upper limb; RLL: right lower limb; LLL: left lower limb; PAD: peripheral arterial disease; AF: atrial fibrillation.

**Figure 2:**
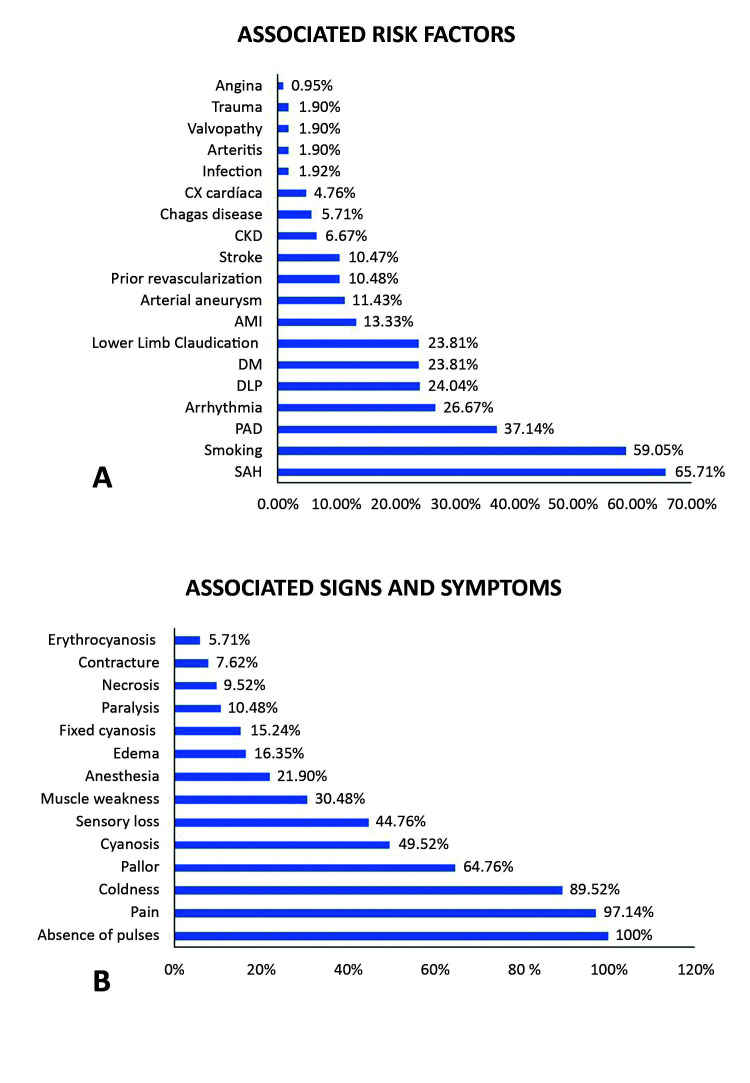
A) Associated risk factors; B) Associated signs and symptoms.

DLP: dyslipidemia; PAD: peripheral arterial occlusive disease; SAH: systemic arterial hypertension; CKD: chronic kidney disease; AMI: acute myocardial infarction; DM: diabetes mellitus.

**Figure 3:**
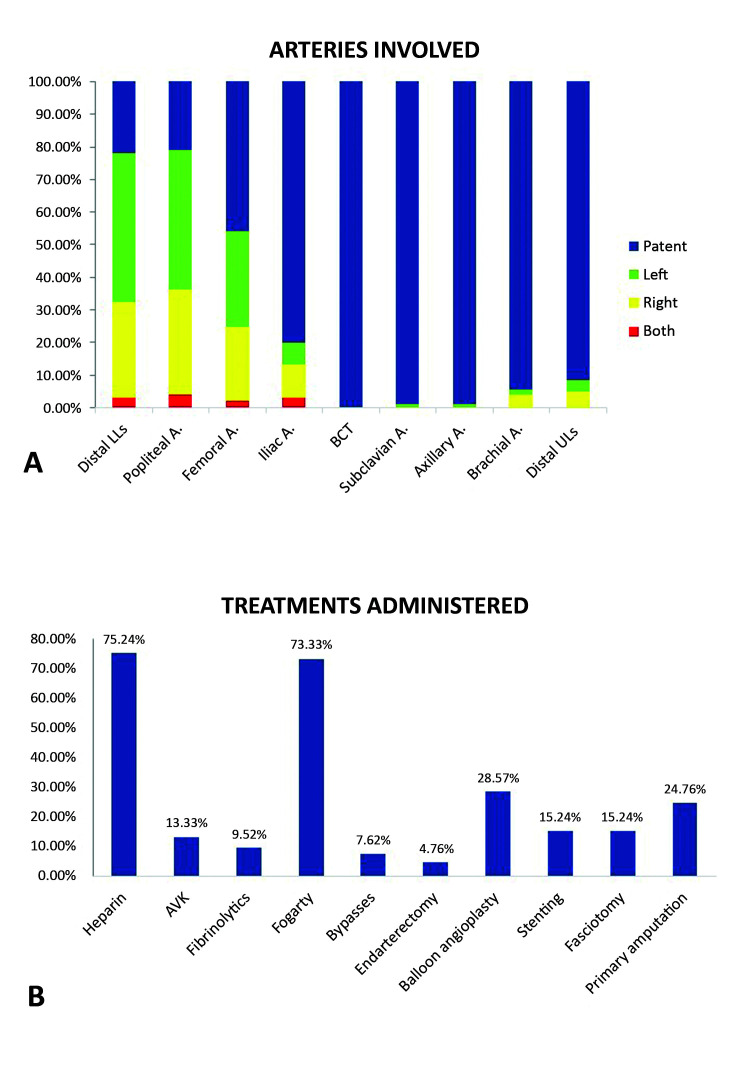
Arteries occluded and treatments administered.

Figure 2 illustrates the frequencies of the main risk factors and signs and symptoms found, showing that hypertension was the most common risk factor (65.71%) (Figure 2A) and that pain was a symptom that was present in almost all of the cases (97.14%) (Figure 2B). Figure 3 illustrates the arteries involved and the treatments administered, showing that the main arteries occluded were arteries of the leg (tibial and fibular) (78.1%), the superficial femoral-popliteal segment (79.05%) and the common femoral artery (54.29%) (Figure 3A). In turn, UFH was used alone or in combination with other treatments in 75.23% of cases and thromboembolectomy with Fogarty catheter was used in 73.33% of cases (Figure 3B).

LLs = lower limbs; A. = artery; BCT = brachiocephalic trunk; ULs = upper limbs; AVK = antivitamin K medications (Warfarin).

**Figure 4:**
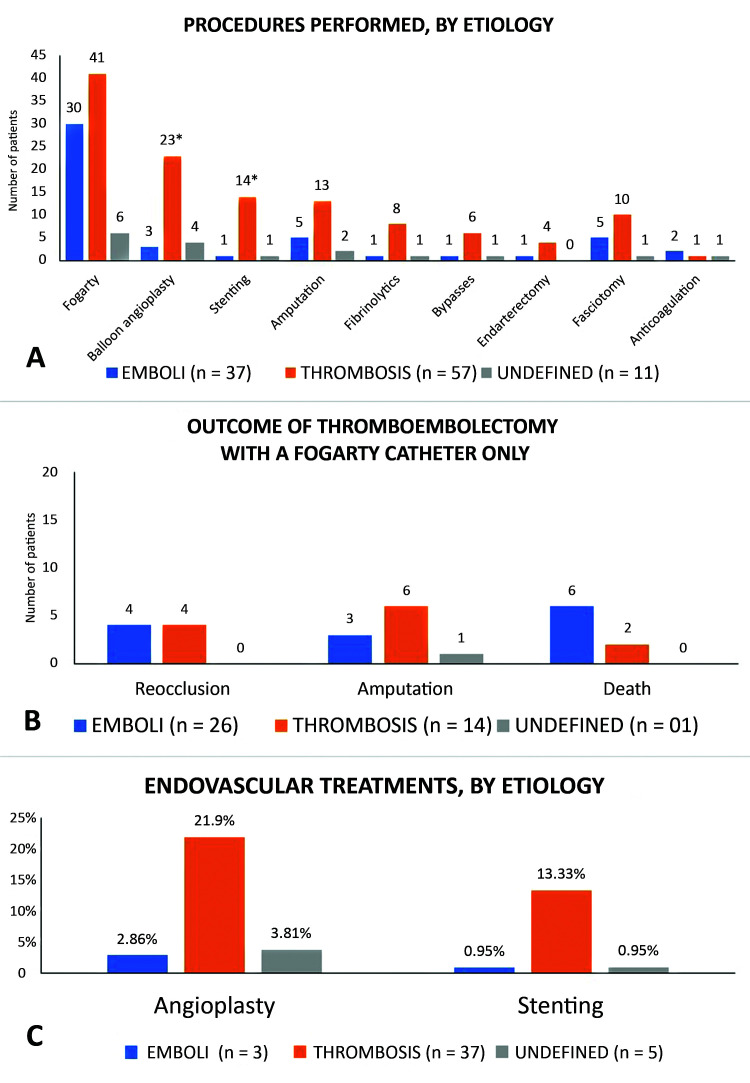
Treatments administered, by etiology. A) Procedures performed, by etiology; B) Outcome of thromboembolectomy with a Fogarty catheter only; C) Endovascular treatments, by etiology.

Figure 4 illustrates certain details of the surgical procedures employed in this study. It is notable that fasciotomy was required in 15.2% of cases and that anticoagulation as sole treatment was only used in 4 patients (3.81%) (Figure 4A). Thromboembolectomy with a Fogarty catheter alone was employed in 41 cases and the association with amputation was more prevalent in thrombotic cases, except for a single undefined case who underwent amputation (Figure 4B). Angioplasty was needed in 29 cases and stenting was performed in 16 cases, predominantly in the thrombosis group (p < 0.05) (Figure 4C). Reocclusion after endovascular treatment occurred in two balloon angioplasties and three stenting cases, all in group 2 – thrombosis.

**Figure 5:**
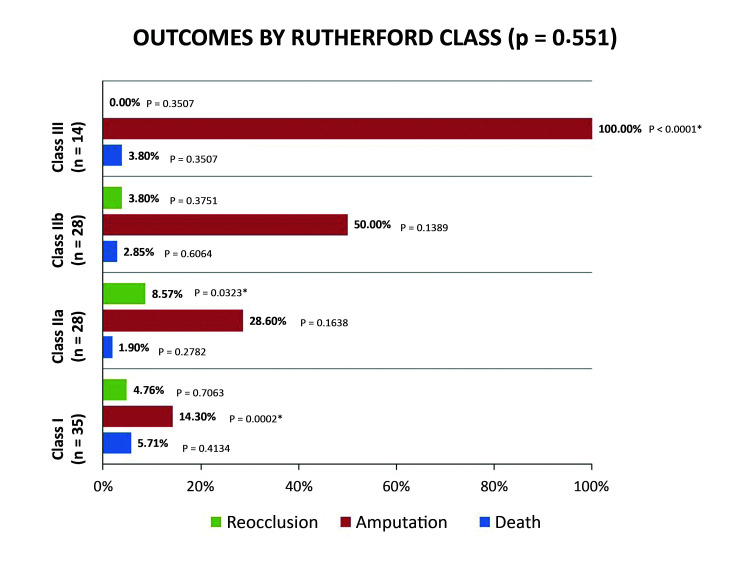
Outcomes by Rutherford class.

Figure 5 illustrates correlations between outcomes and Rutherford classifications. Reocclusion occurred in a higher proportion of class IIa patients (8.57%), with a statistically significant difference in relation to the other classes (p = 0.0323). Amputation was least frequent in class I (14.3%; p = 0.0002) and was most frequent (100%) in class III patients (p < 0.0001). There were no statistically significant differences between classes in terms of death within 30 days. Table 3 lists data found in the literature on demographics, etiology, and arterial restorations, for comparison with the data reported in the present study.

**Table 3:** Demographic data, etiology, and arterial revascularization in the literature compared with data in the present article.

**Author, year of publication**	**Sample (n)**	**Mean age (years)**	**Male/female (%)**	**Thrombosis**	**Emboli**	**Fogarty**	**Total mortality**
Yeager et al.^15^	74	63	95/5	91.9%	8.1%	12.0%	15% (30 d) 49% (36 m)
Davies et al.^14^	77	74	54.5/45.5	41.5%	41.5%	32.5%	26% (30 d)
Comerota et al.^20^	124	66.5	68/32	100% (grafts)	-	30%	8.8% (12 m) All lysis
Aune & Trippestad^17^	372	73	142/130	80	192	58%	17% emboli and 14% thrombosis
Borioni et al.^16^	66	-	-	-	-	100%	32.3% (30 d)
Antusevas & Aleksynas^12^	142	76	74/68	66	76	100% emboli and 45% thromboses	4.2%
Eliason et al.^24^	23,268	71	46/54	-	-	47.3%	9%
Eliason et al.^24^	105	62	57/43	-	-	72.4%	12%
Karapolat et al.^5^	730	61.5	58.4/41.6	15.1%	82.5%	2.5%	3.7%
Comerota et al.^21^	174	63	72.5/27.5	100%	-	0%	6% (12 m)
Present article, 2020	105	69.5	65.7/34.3	54.3%	35.2%	73.3%	14.6%

d = days; m = months.

## DISCUSSION

The patient sample in this study is similar to records in the literature with relation to age, sex, etiology, and use of arterial thromboembolectomy with a Fogarty catheter as the most common type of treatment. Mortality rates and follow-up periods are variable in the literature and in the present study mortality was assessed at 30 days. According to palpation of pulses, arterial involvement was most common in the femoropopliteal segment (79.0%) and the tibial arteries (78.1%), which is also seen in the literature.^12^

The risk factors in this sample were similar to those observed by Donato et al.^13^ in 322 patients with AAO, but the risk profiles varied as a function of specific characteristics of the study population. The reduced frequency of valve disease caused by rheumatic fever and the increasing use of oral anticoagulants in patients with AF has reduced the occurrence of embolic AAO, but the incidence of PAD is increasing as life expectancy increases.^3^ Differential diagnosis between emboli and thrombosis can be difficult to determine in around 10 to 15% of cases, which was confirmed in the present study (10.45% of cases).^3^ In cases in which etiology can be confirmed, presence of AF was the most frequent cause of AAO of embolic origins and it is likely that this is a result of ineffective anticoagulant treatment or failure to comply with anticoagulant treatment. The frequencies of embolic or thrombotic etiology vary in accordance with the regional population characteristics of each study and with the manner in which diagnosis is confirmed.^14^^,^^15^

In the present study, approximately two thirds of the patients had advanced ischemia (Rutherford classes II and III), reflecting a more serious clinical situation (Table 2 and Figure 5). The surgical treatment predominantly employed was thromboembolectomy with a Fogarty catheter, irrespective of etiology, as described by Borioni et al.^16^ In cases with thrombotic etiology, atheromatous plaques make the technique more difficult, but when it is successful, it can remove the secondary thrombus, enabling the state of the artery wall to be assessed using routine intraoperative arteriography. Thromboembolectomy can ameliorate an acute ischemic state, enabling supplementary procedures to be performed using endovascular techniques (hybrid) or surgical methods (endarterectomies or bypasses), reducing the indications for primary endovascular treatment.

The use of thromboembolectomy with a Fogarty catheter in the majority of cases (73.3%) was associated with similar reocclusion rates for embolic and thrombotic etiologies and the complications of thromboembolectomy in isolation were similar to those seen in the literature.^8^ However, because of the greater complexity of treatment and higher rate of comorbidities among thrombotic cases, they appear to be more subject to reocclusion than embolic cases, as shown by Mandelli et al.,^8^ who reported rates of 15.5% vs. 8.9%, respectively, and sometimes supplementary procedures are needed intraoperatively.

Some authors prefer to treat thrombotic AAO using bypasses or endovascular techniques (pharmacological or mechanical thrombolysis combined or not with angioplasty), but it should be noted that pharmacological thrombolysis via catheter with no use of mechanical devices is associated with a hemorrhage risk that is related to the dose of thrombolytics used and may allow ischemic complications to deteriorate because of the time taken for administration, limiting its use to Rutherford classes I/IIA.^3^^,^^17^ When mechanical devices are used in conjunction, they reduce both time taken for revascularization and exposure to the fibrinolytic agent, but raise the cost of the procedure, which limits their use with patients treated on the SUS.

In the Rutherford III cases included in the present study, thromboembolectomy was used to alleviate the ischemic state and/or restore blood flow through collateral arteries, with the objective of achieving a more distal level of amputation. However, in situations of irreversible ischemia, primary amputation was chosen.^3^

Other authors have described chemical thrombolysis as superior to open surgery.^1^^,^^14^^,^^18^^,^^19^ Comerota et al.^20^ compared fibrinolytic treatment to construction of a new bypass in a sample of cases of occlusion of synthetic or autologous bypass grafts comprising 48% acute occlusions and 52% chronic occlusions. They observed a 39% failure rate with fibrinolytic treatment, concluding that surgical treatment with bypass was more effective at 30 days (p = 0.023) and 1 year (p = 0.04), with 84% limb salvage at 12 months (p = 0.026). The same authors analyzed 174 cases of AAO (thrombotic PAD or bypass occlusion, with onset of symptoms less than 2 weeks previously) and achieved a 59% success rate with thrombolytic treatment (lysis exceeding 50%), but with a rate of severe adverse events that varied from 24 to 29%.^21^ Hemorrhage is a worrying complication related to use of thrombolytics, including intracranial hemorrhages in 1 to 2% of cases.^3^^,^^22^ Additionally, use of this technique can be restricted by the cost of medications and multi perforated catheters and by the need for intensive care, laboratory tests, and sequential angiographs.^23^ A recent systematic review study did not find evidence in favor of pharmacological thrombolysis compared with conventional surgery and showed that conventional surgery should be preferred on the basis of limb salvage and mortality at 30 days, 6 months, and 1 year.^24^

Anticoagulation with UFH avoids progression of secondary thrombi, preserving collateral circulation and improving prognosis of arterial restorations, so it is recommended as soon as a diagnosis of AAO is made (100-150 UI/kg).^15^^,^^25^ Heparinization should be maintained during the postoperative period after embolic cases, preventing recurrence. However, platelet antiaggregation is recommended for thrombotic cases revascularized using bypasses or endovascular techniques. The patients in the present study were often referred without anticoagulation, compromising their prognosis. However, anticoagulation was prescribed at patient admission in the majority of cases (75.24%). Blaisdell et al.^26^ recommended primary amputation for nonviable limbs and anticoagulant therapy alone for treatment of viable limbs, which was the conduct employed in four patients in this sample who had viable limbs and no possibility of surgery. Treatment with UFH only has also been related to less occurrence of compartment syndrome and need for fasciotomy,^25^ which was performed in 15.24% of cases in the present sample. It should be noted that use of non-synthetic heparins can be related to heparin-induced thrombocytopenia, which is a rare, but severe, adverse event.^27^

Despite the progress that has been achieved, amputation rates in the range of 10 to 30% and perioperative mortality (30 days) of around 15% are still very high.^13^ Although the majority of the patients treated at the service have a more severe clinical classification, the rates observed in this study are comparable to data in the literature.^28^ In a large multicenter study published by Eliason et al.^25^ (n = 23,168), 47.3% underwent thromboembolectomy, 12.7% had amputations, 10.6% underwent thrombolysis, and 12.5% were treated with angioplasty, with a 9.3% intrahospital mortality rate. A subset of the same study (University of Michigan, n = 105) observed 14.3% amputations, 72.4% thromboembolectomies, 24.8% fasciotomies, 38.1% thrombolysis, and 7.7% bypasses, with 11.4% mortality, demonstrating the heterogeneous nature of results that are dependent on factors intrinsic to each population. These authors found that choosing embolectomy was associated with lower rates of amputations and mortality.^25^ However, compartment syndrome and the need for fasciotomy is a parameter with greater variation between studies. It is often associated with duration of ischemia and with embolic etiology, but in the present study it was more common among thrombotic cases, probably because of the prolonged ischemia time.

In a study by Davies et al.,^14^ intra-arterial thrombolysis was associated with excellent results, with an 85% limb salvage rate, 9% amputation, and 6% mortality, whereas thromboembolectomy results were 71% limb salvage, 3% amputation, and 26% mortality (at 30 days). Comerota et al.^21^ observed 59% success of thrombolytic therapy in patients with PAD or occluded bypasses (less than 2 weeks since onset of symptoms) and severe adverse event rates varying from 24 to 29% (n = 174). Davies et al.,^14^ Ouriel et al.,^18^ and a consensus statement^29^ report thrombolysis as potentially advantageous in relation to surgery, for all types of etiology. In contrast, Costantini et al.,^3^ consider that immediate surgical revascularization is indicated for critically ischemic limbs and catheter-directed thrombolysis should be used for cases that are not clinically threatened by ischemia.

Yeager et al.^15^ treated 86% of their patients with anticoagulation with UFH, 70% underwent preoperative angiography, 65% underwent revascularization surgery and 12% had thromboembolectomy, with a 70% limb salvage rate and 15% mortality (at 1 month), with no relationship with reperfusion of the limb.

This study has the following possible limitations: 1) analysis of just 105 of 288 patient medical records, in a partially retrospective manner and slightly below the ideal sample size calculated; 2) etiologic diagnosis not entirely supported by a diagnostic gold standard; and 3) incomplete laboratory test data.

## CONCLUSIONS

The predominant treatment was thromboembolectomy with a Fogarty catheter, used in 73.3% of cases, used in the majority of cases, either in isolation or in combination with other treatments, irrespective of etiology. It was the exclusive treatment in 41 cases (39.05%): 70.27% of embolic cases, 24.56% of thrombotic cases, and 9.09% of those with undefined etiology. Cases with thrombotic etiology showed a trend towards a greater frequency of reocclusions, but without correspondingly higher amputation rates or in-hospital death.

The rate of major amputation within 30 days after restoration was 19.05%, similar to in the literature (16 to 30%),^7^ and mortality within 30 days was 13.34%, lower than rates in the literature (18 to 25%).^7^ Thus, arterial thromboembolectomy with a Fogarty catheter, in isolation or associated with other treatments, was associated with comparable rates of amputation and complications to those found in the literature.
