# Supplementary material for: Results of treatment of acute occlusions of limb arteries at a university hospital - retrospective study
Source: J Vasc Bras. 2020 Nov 16;19:e20200031. doi: 10.1590/1677-5449.200031 (PMC8218016; doi:10.1590/1677-5449.200031)
Supplement: Translation

# Translation: Resultados do tratamento das oclusões arteriais agudas de membros em hospital universitário – estudo retrospectivo

**Authors:** Caroline Teodoro, Matheus Bertanha, Flavia Potsch Camara Mattos Girard, Marcone Lima Sobreira, Ricardo de Alvarenga Yoshida, Regina Moura, Rodrigo Gibin Jaldin, Winston Bonetti Yoshida

**Affiliations:** 1 Cirurgia e Ortopedia, Universidade Estadual Paulista “Júlio de Mesquita Filho” (UNESP), Botucatu, SP, Brasil.

**Keywords:** embolectomia com balão, isquemia, extremidade inferior, extremidade superior

## Abstract

**Contexto:**

As oclusões arteriais agudas (OAA) de membros vêm crescendo paralelemente com a longevidade da população.

**Objetivos:**

O objetivo deste estudo foi avaliar fatores de risco, salvamento de membros e sobrevida dos pacientes com OAA tratados em instituição universitária.

**Métodos:**

Este é um estudo coorte retrospectivo de pacientes consecutivos. Os desfechos incluíram: sucesso técnico, sintomas, comorbidades, categoria Rutherford, artérias acometidas, complicações pós-operatórias, taxa de salvamento de membros em 30 dias e óbitos.

**Resultados:**

Avaliou-se 105 prontuários, havendo predomínio do sexo masculino (65,7%) e idade entre 46 a 91 anos. As etiologias identificadas foram trombóticas (54,3%), embólicas (35,2%) e indefinidas (10,5%). Cerca de dois terços apresentavam-se nas Categorias II e III de Rutherford. Os sintomas associados encontrados foram dor (97,1%), esfriamento (89,5%), palidez (64,7%), parestesias (44,7%), paralisias (30,5%), anestesias (21,9%), edema (21,9%) e cianose (15,2%); e as comorbidades associadas observadas foram hipertensão (65,0%), tabagismo (59,0%), arritmias (26,6%), dislipidemias (24,0%) e diabetes (23,8%). O segmento femoral superficial-poplíteo-distal foi o mais acometido (80%). A tromboembolectomia com cateter Fogarty foi realizada em 73,3% dos casos (81,0% nas embolias, 71,9% nas tromboses e 54,5% nos indefinidos), sendo isoladamente em 41 pacientes (39,05%), nos quais ocorreram 11 reoclusões, 20 amputações e 14 óbitos. A reoclusão arterial foi mais frequente nas tromboses (12,9%; p = 0,054). Até 30 dias após tratamento, o óbito total foi de 14,6% e a amputação maior foi de 19,8%, sendo menos frequente na Classe I Rutherford (p = 0,0179).

**Conclusão:**

O tratamento da OAA feito prioritariamente por meio de tromboembolectomia com cateter Fogarty, isolado e/ou associado, proporcionou taxas de amputação e complicações compatíveis com as apresentadas na literatura e progressivamente menores nas categorias Rutherford menos avançadas.

## INTRODUÇÃO

A oclusão arterial aguda (OAA) é definida como um súbito decréscimo da perfusão sanguínea, que ameaça a viabilidade do membro^1^. Os sintomas dolorosos em geral são abruptos e variam desde claudicação intermitente de início súbito ou piora da claudicação, dor em repouso, frialdade (poiquilotermia), parestesias, fraqueza muscular ou paralisia. No exame físico, os pulsos estão ausentes distais à oclusão, com esfriamento, palidez ou cianose da pele e perda de sensibilidade do membro acometido^2^. Sem intervenção vascular precoce em tempo hábil, a evolução, na maioria dos casos, tem um prognóstico reservado quanto ao salvamento do membro, podendo-se instaurar isquemia irreversível do membro com a necessidade de amputações.

A sua incidência varia entre 14/100.000 habitantes e 17/100.000 habitantes ou 1,5 casos por 10.000 pessoas por ano^1^^,^^3^^,^^4^ e vem crescendo com o aumento da longevidade da população. As causas mais frequentes são as embolias oriundas de alterações cardíacas ou aórticas e tromboses de placas ateroscleróticas, consequentes de complicações aterotrombóticas nas artérias periféricas ou por oclusões de restaurações arteriais, além dos traumas em pacientes não ateroscleróticos, especialmente os iatrogênicos^3^.

A restauração do fluxo sanguíneo pode ser feita por técnicas endovasculares (trombólise mecânica ou farmacológica, angioplastias e stents) ou por meio de cirurgias abertas, como tromboembolectomias com cateter Fogarty, pontes (*bypass*), endarterectomias com ou sem remendos arteriais ou técnicas híbridas^1^. Os pacientes com embolias femorais e poplíteas têm maiores taxas de amputações, diretamente relacionadas com o tempo entre a oclusão e o tratamento^5^. Nos casos de tromboses arteriais relacionadas à doença arterial periférica (DAP), o tratamento da placa de ateroma complicada subjacente ao trombo é fundamental para manter o sucesso na revascularização.

Um dos métodos mais consagrados de tratamento é a tromboembolectomia com cateter balão complacente, introduzido por Fogarty et al.^6^ (1963), sendo mais efetivo nas embolias do que em outras etiologias^1^^,^^7^. Embora essas técnicas tenham revolucionado o tratamento das OAAs, ainda ocorrem muitas complicações, e as taxas de amputações variam de cerca de 6 a 30% e a de mortalidade, de 18 a 25%^1^^,^^3^.

Há poucas informações sobre resultados e evolução de pacientes com OAA tratados pelo Sistema Único de Saúde (SUS) no Brasil. Nas publicações nacionais, sobressaem estudos com uso de fibrinolíticos^7^^-^10, embora esse tratamento não seja facilmente disponível em hospitais vinculados ao SUS. Não se encontrou na literatura qualquer estudo brasileiro com os resultados do uso sistemático e prioritário de tromboembolectomia com cateter Fogarty nas OAAs de extremidades independentemente da etiologia, o que justificou a realização do presente estudo.

## OBJETIVO

O objetivo deste estudo foi avaliar retrospectivamente os casos de OAAs tratados em serviço público universitário de referência em alta complexidade cardiovascular do SUS, analisando os fatores de risco, os resultados de salvamento de membros e a sobrevida perioperatória desses pacientes.

## MÉTODOS

Foi realizado um estudo de coorte retrospectivo de série consecutiva de casos. Foram incluídos os pacientes com diagnóstico de OAA de membros inferiores ou superiores que receberam tratamento no período entre 2012 e 2017, através da análise de dados de prontuários eletrônicos de um único centro universitário. Foram excluídos pacientes menores de 18 anos, com oclusões de derivações arteriais, gestantes, portadores de neoplasias e arterites e prontuários incompletos. Este trabalho foi aprovado pelo Comitê de Ética em Pesquisa local (Parecer: 737.804).

Em todos casos, foi preenchido prospectivamente um formulário específico para OAA, cujos dados faltantes foram completados por revisão retrospectiva dos prontuários. Foram tabulados dados demográficos, fatores de risco, sinais e sintomas, categorias da classificação clínica de Rutherford para o grau de isquemia do membro acometido (classificação da *Society of Vascular Surgery/The International Society of Cardiovascular Surgery*)^11^ (Tabela 1), tratamentos efetuados e suas principais complicações (amputações, reoclusões, necessidade de fasciotomia, insuficiência renal, complicações pulmonares e cardíacas, amputações e mortalidade operatória).

**Tabela 1:** Classificação clínica de acordo com a evolução da isquemia do membro acometido, adaptado de Rutherford (classificação da *Society of Vascular Surgery/The International Society of Cardiovascular Surgery*)^3^.

**Classificação**	**Sinais clínicos e exame ao Doppler**
Classificação I – Membro viável	Ausência de sinais neurológicos, som arterial audível ao Doppler.
Classificação II – Viabilidade ameaçada – isquemia reversível	IIa – Marginalmente ameaçado: parestesia, sem som arterial ao Doppler, som venoso presente.
	IIb – Ameaça imediata: algum grau de paresia, sem som arterial ao Doppler, som venoso presente.
Classificação III – Membro inviável	Paralisia, contratura, sem som arterial ou venoso ao Doppler.

Os pacientes foram divididos em três grupos, de acordo com a etiologia das OAAs: grupo 1 – embolias; grupo 2 – tromboses; e grupo 3 – indefinido. Foram considerados para esse diferencial: o início do quadro como agudo ou gradual; dor como intensa ou moderada; antecedente de claudicação intermitente ausente ou presente; arritmia cardíaca presente ou ausente; pulsos contralaterais presentes ou ausentes; achados intraoperatórios compatíveis com embolia ou trombose^1^. Foram considerados como indefinidos os pacientes que não se encaixaram nos critérios característicos de embolia ou trombose.

O protocolo de tratamento realizado no hospital foi o uso prioritário da tromboembolectomia com cateter Fogarty para alívio imediato da isquemia aguda, tanto nas embolias como em casos de trombose. A via preferencial para o acesso cirúrgico foi a dissecção e exposição, conforme o caso, das artérias femoral comum, femoral superficial ou braquial, complementados por acessos distais quando necessário. O protocolo incluiu a realização rotineira de arteriografia intraoperatória após a tromboembolectomia para a avaliação do resultado e identificação de outras estratégias durante a cirurgia.

A heparina não fracionada (HNF) foi utilizada no pós-operatório dos casos embólicos para prevenção de recorrência. Nos casos trombóticos tratados por procedimentos endovasculares ou pontes, o uso de dupla antiagregação plaquetária foi o tratamento indicado. Nos casos Rutherford III, a tromboembolectomia com cateter Fogarty foi indicada em algumas situações para aliviar o quadro isquêmico e/ou para restaurar o fluxo sanguíneo em artérias colaterais a fim de tentar realizar uma amputação mais distal e/ou preservação osteoarticular, visando melhor reabilitação. Os desfechos primários foram: óbito, reoclusão arterial e necessidade de amputação maior (transtibial e/ou transfemoral ou de membro superior).

## CASUÍSTICA

A amostra foi de conveniência, avaliando-se todos os casos consecutivos do período que preencheram os critérios de inclusão. Tomando-se por base informações da literatura entre 18 a 25% de mortalidade (considerando o valor mediano de 22%) e esperando-se diminuição dessa taxa para 15%, o tamanho amostral foi calculado em 135 pacientes, com 80% do poder do teste e 5% de nível de significância.

## ANÁLISE ESTATÍSTICA

Foram calculadas estatísticas descritivas para as variáveis quantitativas, as quais foram estratificadas pelo diagnóstico final (embolia, trombose e indefinido). A comparação entre as médias para os diagnósticos foi feita utilizando a análise de variância (ANOVA), seguida do teste de Tukey. As comparações de dados contínuos entre os momentos pré e pós-operatórios foram feitas pelo teste *t* de Student, pareado para cada diagnóstico. Para as variáveis categorizadas, as associações com o diagnóstico final foram avaliadas através do teste qui-quadrado ou de Fisher. Foi considerado o nível de significância de 5% ou o p-valor correspondente. Todas as análises foram feitas pelo programa SAS for Windows, v.9.4 (SAS Institute Inc., Carolina do Norte, EUA).

## RESULTADOS

Dos 288 registros de OAA encontrados, 183 foram excluídos devido aos fatores explicitados na Figura 1. Portanto, foram analisados 105 prontuários. A Tabela 2 apresenta os dados demográficos e desfechos de acordo com a etiologia (por grupos). A etiologia trombótica foi a mais prevalente (54,3%), seguida pela etiologia embólica (35,2%) e de origem indefinida (10,5%), sem diferença entre os sexos. A etnia branca foi a mais frequente (85,7%), e os membros inferiores foram os mais acometidos (89,5%). A classificação de Rutherford teve menor número absoluto de pacientes no grupo III, porém sem diferença estatística entre os grupos. Em relação aos desfechos reoclusão, amputação e óbito em 30 dias, não houve diferença estatística entre os grupos. A fibrilação atrial (FA) esteve mais presente associada estatisticamente ao grupo 1 – embolia (p = 0,0001), e a presença de aneurisma de artéria poplítea, DAP e tabagismo foram estatisticamente mais frequentes no grupo 2 – trombose (p = 0,0138, p = 0,0125 e p = 0,0161, respectivamente).

**Figura 1:** Fluxograma de inclusão dos pacientes selecionados para este estudo.

**Tabela 2:** Dados demográficos e principais desfechos, de acordo com a etiologia da oclusão arterial aguda (OAA).

		**Embolia (n = 37)**	**Trombose (n = 57)**	**Indefinido (n = 11)**	**Total (n = 105)**	**Valor de p**
Idade (anos)	Máxima	91	87	90	91	-
	Mínima	48	47	52	46	-
	Média	70	69	73	69,5	-
Sexo	Masculino	25 (23,81%)	37 (35,24%)	7 (6,67%)	69 (65,71%)	0,9542
	Feminino	12 (11,43%)	20 (19,05%)	4 (3,81%)	36 (34,29%)	
Etnia	Caucasiano	28 (26,67%)	53 (50,48%)	9 (8,57%)	90 (85,7%)	0,0164
	Outra	9 (8,56%)	4 (3,81%)	2 (1,9%)	15 (14,3%)	
Membro	MSD	4 (3,81%)	0	1 (0,95%)	5 (4,76%)	
	MSE	2 (1,9%)	1 (0,95%)	1 (0,95%)	4 (3,81%)	
	MID	13 (12,38%)	19 (18,1%)	7 (6,67%)	39 (37,14%)	
	MIE	16 (15,24%)	36 (34,29%)	2 (2,86%)	55 (52,38%)	
	Ambos os membros inferiores	2 (1,9%)	0	0	2 (1,9%)	
Classificação Rutherford^11^	I	12 (11,43%)	19 (18,1%)	4 (3,81%)	35 (33,3%)	0,9709
	IIa	11 (10,48%)	15 (14,29%)	2 (1,9%)	28 (26,7%)	0,7460
	IIb	11 (10,48%)	15 (14,29%)	2 (1,9%)	28 (26,7%)	0,7460
	III	3 (2,86%)	8 (7,62%)	3 (2,86%)	14 (13,3%)	0,2531
Reoclusão		4 (3,81%)	13 (12,38%)	0	17 (16,19%)	0,0546
Amputação primária		10 (9,52%)	12 (11,43%)	4 (3,81%)	26 (24,76%)	0,5175
Mortalidade 30 em dias		6 (5,71%)	7 (6,67%)	1 (0,95%)	14 (13,34%)	0,7877
Fatores de risco	Aneurisma poplíteo	0	9 (8,6%)	3 (2,9%)	12 (11,4%)	0,0138
	DAP	7 (6,7%)	28 (26,7%)	4 (3,8%)	39 (37,1%)	0,0125
	Tabagismo	15 (14,3%)	40 (38,1%)	7 (6,7%)	62 (59,0%)	0,0161
	FA	19 (18,1%)	1 (0,95%)	1 (0,95%)	21 (20%)	0,0001

MSD: membro superior direito; MSE: membro superior esquerdo; MID: membro inferior direito; MIE: membro inferior esquerdo; DAP: doença arterial periférica; FA: fibrilação atrial.

A Figura 2 apresenta os principais fatores de risco e os sinais e sintomas encontrados, sendo hipertensão o fator de risco mais presente (65,71%) (Figura 2A) e a dor o sintoma presente na quase totalidade dos casos (97,14%) (Figura 2B). A Figura 3 apresenta as artérias acometidas e os tratamentos efetuados, sendo as principais artérias ocluídas as artérias da perna (tibiais e fibular) (78,1%), o segmento femoral superficial-poplíteo (79,05%) e a artéria femoral comum (54,29%) (Figura 3A). A HNF foi empregada isoladamente ou em associação em 75,23% dos casos e a tromboembolectomia com cateter Fogarty, empregado em 73,33% dos casos (Figura 3B).

**Figura 2:** A) Fatores de risco associados; B) Sinais e sintomas associados.

AVC: acidente vascular cerebral; MMII: membros inferiores; DLP: dislipidemia; DAP: doença arterial obstrutiva periférica; HAS: hipertensão arterial sistêmica; DRC: doença renal crônica; IAM: infarto agudo do miocárdio; DM: diabetes melito.

**Figura 3:** Artérias ocluídas e tratamentos efetuados.

MMII = membros inferiores; A. = artéria; TBC = tronco braquiocefálico; MMSS = membros superiores; AVK = medicamentos antivitamina K (Varfarina).

A Figura 4 apresenta algumas particularidades dos procedimentos cirúrgicos empregados neste estudo. Destaca-se que as fasciotomias foram necessárias em 15,2% dos casos e a anticoagulação exclusiva foi realizada em apenas 4 pacientes (3,81%) (Figura 4A). A tromboembolectomia com cateter Fogarty isoladamente foi empregada em 41 casos, sendo associada a amputação mais prevalente em casos trombóticos, excetuando-se o único caso indefinido que sofreu amputação (Figura 4B). Já a angioplastia foi necessária em 29 casos e o implante de stent, em 16 casos, predominantemente no grupo trombose (p < 0,05) (Figura 4C). A reoclusão relacionada ao tratamento endovascular ocorreu em duas angioplastias com balão e em três implantes de stent, todos do grupo 2 – trombose.

**Figura 4:** Tratamentos realizados de acordo com a etiologia. A) Procedimentos realizados por etiologia; B) Evolução da tromboembolectomia com cateter Fogarty exclusivo; C) Tratamentos endovasculares por etiologia.

A Figura 5 apresenta a correlação entre os desfechos e a classificação de Rutherford. Quanto a reoclusão, ela ocorreu em maior número de pacientes da classe IIa (8,57%), sendo estatisticamente significante em relação às outras classes (p = 0,0323). Quanto à amputação, ocorreu menos frequentemente na classe I (14,3%; p = 0,0002) e foi maior (100%) nos pacientes da classe III (p < 0,0001). Quanto ao óbito em até 30 dias, não houve diferença estatisticamente significativa entre as classes. A Tabela 3 apresenta demográficos, etiologia e restaurações arteriais encontrados na literatura, para comparação com os dados do presente estudo.

**Figura 5:** Desfechos por classes Rutherford.

**Tabela 3:** Dados demográficos, etiologia e restaurações arteriais na literatura em comparação com os do presente artigo.

**Autor, ano**	**Amostra (n)**	**Idade média (anos)**	**Sexo masculino/feminino (%)**	**Trombose**	**Embolia**	**Fogarty**	**Óbito total**
Yeager et al.^15^	74	63	95/5	91,9%	8,1%	12,0%	15% (30 d) 49% (36 m)
Davies et al.^14^	77	74	54,5/45,5	41,5%	41,5%	32,5%	26% (30 d)
Comerota et al.^20^	124	66,5	68/32	100% (enxertos)	-	30%	8,8% (12 m) Todos fibrinólise
Aune & Trippestad^17^	372	73	142/130	80	192	58%	17% embolia e 14% trombose
Borioni et al.^16^	66	-	-	-	-	100%	32,3% (30 d)
Antusevas & Aleksynas^12^	142	76	74/68	66	76	100% embolias e 45% tromboses	4,2%
Eliason et al.^24^	23.268	71	46/54	-	-	47,3%	9%
Eliason et al.^24^	105	62	57/43	-	-	72,4%	12%
Karapolat et al.^5^	730	61,5	58,4/41,6	15,1%	82,5%	2,5%	3,7%
Comerota et al.^21^	174	63	72,5/27,5	100%	-	0%	6% (12 m)
Presente artigo, 2020	105	69,5	65,7/34,3	54,3%	35,2%	73,3%	14,6%

d = dias; m = meses.

## DISCUSSÃO

A casuística deste estudo foi similar aos registros da literatura com relação a idade, sexo, etiologia e a realização de tromboembolectomia arterial com uso do cateter Fogarty como forma de tratamento mais frequente. A taxa de óbitos e o período de seguimento foram variáveis na literatura, sendo que o presente estudo avaliou esse desfecho em até 30 dias. Pela palpação dos pulsos, o acometimento arterial foi mais frequente no segmento fêmoro-poplíteo (79,0%) e nas artérias tibiais (78,1%), o que também é verificado na literatura^12^.

Os fatores de risco da presente casuística foram similares aos encontrados por Donato et al.^13^ em 322 pacientes com OAA, mas os perfis de risco variaram em função de características peculiares da população estudada. A diminuição da frequência de valvulopatias causadas por febre reumática e o crescente uso de anticoagulantes orais em pacientes com FA têm reduzido as ocorrências de OAA embólicas, porém a incidência de DAP vem crescendo pelo aumento da expectativa de vida^3^. O diagnóstico diferencial entre embolia e trombose pode ser difícil de estabelecer em cerca de 10 a 15% dos casos, o que é corroborado no presente estudo (10,45% dos casos)^3^. Nos casos em que a etiologia pôde ser confirmada, a presença de FA foi a causa mais frequente de OAA de origem embólica, sendo provável que ocorra por ineficácia ou falta de aderência ao tratamento anticoagulante. As frequências da etiologia embólica ou trombótica variam conforme as características populacionais regionais de cada estudo e a forma de confirmação desse diagnóstico^14^^,^^15^.

No presente estudo, aproximadamente dois terços dos pacientes apresentavam isquemia avançada (classes Rutherford II e III), refletindo uma situação clínica mais grave (Tabela 2 e Figura 5). O tratamento cirúrgico predominantemente empregado foi a tromboembolectomia com cateter Fogarty, independentemente da etiologia, como apresentado por Borioni et al.^16^ Nos casos de etiologia trombótica, a técnica é dificultada pela presença da placa de ateroma, mas, quando bem-sucedida, pode remover o trombo secundário, permitindo a avaliação das condições da parede arterial por meio de arteriografia rotineira no intraoperatório. A tromboembolectomia pode amenizar um quadro isquêmico agudo, possibilitando a realização de procedimentos complementares por técnicas endovasculares (híbridos) ou cirúrgicas (endarterectomias ou pontes), reduzindo a indicação do tratamento endovascular primário.

A opção pela tromboembolectomia com cateter Fogarty para a maioria dos casos (73,3%) apresentou taxas de reoclusão similares entre as etiologias embólica e trombótica e as complicações da tromboembolectomia isolada foram semelhantes às encontradas na literatura^8^. No entanto, os casos trombóticos, em decorrência da maior complexidade do tratamento e comorbidades, parecem estar mais sujeitos à reoclusão que os embólicos, segundo Mandelli et al.^8^, que apresentaram taxas de 15,5% vs. 8,9%, respectivamente, por vezes necessitando de procedimentos complementares no intraoperatório.

Alguns autores preferem o tratamento de OAA trombótica por pontes ou técnicas endovasculares (trombólise farmacológica ou mecânica associada ou não a angioplastia), mas deve-se observar que a trombólise farmacológica por cateter não associada a dispositivos mecânicos apresenta risco hemorrágico relacionado à dose de trombolíticos e pode deteriorar o quadro isquêmico pelo tempo de aplicação, limitando-se às classes Rutherford I/IIA^3^^,^^17^. Quando associada a dispositivos mecânicos, ela reduz o tempo de revascularização e a exposição ao agente fibrinolítico, porém eleva o custo do procedimento e limita sua aplicação aos pacientes do SUS.

Nos casos da classe Rutherford III, no presente estudo, a tromboembolectomia foi usada para aliviar o quadro isquêmico e/ou restaurar o fluxo sanguíneo em artérias colaterais, objetivando melhorar o nível de uma amputação. Entretanto, para a situação de isquemia irreversível, a amputação primária foi a escolha^3^.

Outros autores têm apresentado a trombólise química como superior à modalidade cirúrgica aberta^1^^,^^14^^,^^18^^,^^19^. Comerota et al.^20^ compararam o tratamento fibrinolítico à confecção de nova ponte para casos de oclusões de pontes sintéticas ou autólogas, sendo 48% de oclusões agudas e 52% de oclusões crônicas. Observaram 39% de insucesso no tratamento fibrinolítico, concluindo que o tratamento cirúrgico por ponte foi mais eficaz em 30 dias (p = 0,023) e 1 ano (p = 0,04), com 84% de salvamento de membro em 12 meses (p = 0,026). O mesmo autor avaliou 174 casos de OAA (DAP trombótica ou oclusão de pontes, com sintomas há menos de 2 semanas) e obteve 59% de sucesso na terapia trombolítica (lise superior a 50%), porém com taxa de eventos adversos graves variando de 24 a 29%^21^. A hemorragia é uma complicação preocupante relacionada ao uso de trombolíticos, incluindo a hemorragia intracraniana em 1 a 2% dos casos^3^^,^^22^. Além disso, essa técnica pode ser limitada pelos custos dos medicamentos e dos cateteres multiperfurados e pela necessidade de terapia intensiva, exames laboratoriais e angiografias sequenciais^23^. Um trabalho recente de revisão sistemática não apontou a existência de evidência favorável a trombólise farmacológica em comparação com a cirurgia convencional e mostrou que a cirurgia convencional deve ser a preferida em termos de salvamento de membro e mortalidade aos 30 dias, 6 meses e 1 ano^24^.

A anticoagulação com HNF evita a progressão de trombos secundários, preservando a circulação colateral e melhorando o prognóstico da restauração arterial, sendo recomendada tão logo o diagnóstico de OAA seja feito (100-150 UI/kg)^15^^,^^25^. A heparinização deve ser mantida no pós-operatório dos quadros embólicos, prevenindo a recorrência; porém, nos casos trombóticos revascularizados por pontes ou técnicas endovasculares, preconiza-se a antiagregação. No presente estudo, frequentemente os pacientes eram referenciados sem anticoagulação, prejudicando o prognóstico. Entretanto, a anticoagulação foi prescrita na admissão do paciente na maioria dos casos (75,24%). Blaisdell et al.^26^ recomendaram a amputação primária para o membro inviável e a terapia anticoagulante isolada para o tratamento do membro viável, o que foi empregado em quatro pacientes dessa casuística que apresentavam membro viável e impossibilidade cirúrgica. O tratamento apenas com HNF também foi menos relacionado à ocorrência de síndrome compartimental e a menor necessidade de fasciotomias^25^, o que foi realizado em 15,24% dos casos na presente casuística. Ressalta-se que a utilização de heparinas não sintéticas pode estar relacionada a trombocitopenia induzida por heparina, que é um evento adverso raro, porém grave^27^.

Apesar dos progressos, o índice de amputações entre 10 a 30% e mortalidade perioperatória (30 dias) de cerca de 15% ainda são muito expressivos^13^. Não obstante o atendimento majoritário a pacientes com classificação clínica mais grave, os índices deste estudo se equipararam aos da literatura^28^. Em um grande estudo multicêntrico conduzido por Eliason et al.^25^ (n = 23.168), foram feitas 47,3% de tromboembolectomias, 12,7% de amputações, 10,6% de trombólises e 12,5% de angioplastias, relatando 9,3% de mortalidade intra-hospitalar. Um subgrupo desse mesmo estudo (Universidade de Michigan, n = 105) observou 14,3% de amputações, 72,4% de tromboembolectomias, 24,8% de fasciotomias, 38,1% de trombólises e 7,7% de pontes, com mortalidade de 11,4%, demonstrando a heterogeneidade de resultados dependentes de fatores próprios de cada população. Para esses autores, a escolha da embolectomia foi associada com menores taxas de amputações e mortalidade^25^. No entanto, a síndrome compartimental e a necessidade de fasciotomia é um parâmetro mais variável entre os estudos, frequentemente associadas ao tempo de isquemia e à etiologia embólica, mas no presente estudo foi mais frequente nos trombóticos, provavelmente em função de tempo da isquemia prolongada.

No estudo de Davies et al.^14^, a trombólise intra-arterial apresentou ótimos resultados, com 85% de salvamento de membros, 9% de amputações e 6% de óbito, sendo que, para a tromboembolectomia, houve 71% de salvamento de membros, 3% de amputações e 26% de óbito (em 30 dias). Comerota et al.^21^ observaram 59% de sucesso na terapia trombolítica em pacientes com DAP ou oclusão de pontes (menos de duas semanas de sintomas) e eventos adversos graves variando de 24 a 29% (n = 174). Davies et al.^14^, Ouriel et al.^18^ e consensos^29^ relataram a trombólise como potencialmente vantajosa em relação à modalidade cirúrgica, em qualquer etiologia. Porém, para Costantini et al.^3^, a revascularização cirúrgica imediata estaria indicada no membro criticamente isquêmico e a trombólise direcionada por cateter nos casos não clinicamente ameaçados pela isquemia.

Yeager et al.^15^ submeteram 86% dos seus pacientes à anticoagulação com HNF, 70% realizaram angiografia pré-operatória, 65% foram submetidos à cirurgia de revascularização, 12%, à tromboembolectomia, com 70% de salvamento de membros e 15% de óbito (em um mês), sem relação com a reperfusão do membro.

O presente estudo apresenta as possíveis limitações: 1) a avaliação de somente 105 dos 288 prontuários, de forma parcialmente retrospectiva e levemente abaixo da amostra calculada; 2) o diagnóstico etiológico não totalmente respaldado por algum padrão-ouro de diagnóstico; e 3) os dados incompletos para exames laboratoriais.

## CONCLUSÕES

O tratamento predominante foi a tromboembolectomia com cateter Fogarty em 73,3% dos casos, tendo sido realizado isoladamente e/ou em associação na maioria dos casos independentemente da etiologia. Foi o tratamento exclusivo em 41 casos (39,05%), dentre os quais: 70,27% nas embolias, 24,56% nas tromboses e 9,09% nos indefinidos. Os casos de etiologia trombótica tiveram tendência a maior frequência de reoclusões, mas sem correspondentes maiores taxas de amputações e óbito hospitalar.

A amputação maior em até 30 dias após restauração foi de 19,05%, similar à da literatura (16 a 30%)^7^, e a mortalidade em até 30 dias foi de 13,34%, inferior da que é encontrado na literatura (18 a 25%)^7^. Assim, a tromboembolectomia arterial com cateter Fogarty, isolado e/ou associada com outros tratamentos, proporcionou índices de amputação e complicações comparáveis com aos encontrados na literatura.
